# Expansion of the phenotype of lateral meningocele syndrome

**DOI:** 10.1002/ajmg.a.61536

**Published:** 2020-03-06

**Authors:** Gerarda Cappuccio, Diletta Apuzzo, Marianna Alagia, Annalaura Torella, Michele Pinelli, Brunella Franco, Bruno Corrado, Ennio del Giudice, Alessandra D'Amico, Vincenzo Nigro, TUDP, Nicola Brunetti‐Pierri

**Affiliations:** ^1^ Department of Translational Medicine Federico II University Naples Italy; ^2^ Telethon Institute of Genetics and Medicine, Pozzuoli Naples Italy; ^3^ Medical Genetics, Department of Biochemistry, Biophysics and General Pathology University of Campania 'Luigi Vanvitelli' Naples Italy; ^4^ Department of Public Health Federico II University of Naples Naples Italy; ^5^ Department of Advanced Biomedical Sciences Federico II University Naples Italy

**Keywords:** encephalocele, lateral meningocele syndrome, *NOTCH3*

## Abstract

Lateral meningocele syndrome (LMS) is due to specific pathogenic variants in the last exon of *NOTCH3* gene. Besides the lateral meningoceles, this condition presents with dysmorphic features, short stature, congenital heart defects, and feeding difficulties. Here, we report a girl with neurosensorial hearing loss, severe gastroesophageal reflux disease, congenital heart defects, multiple renal cysts, kyphosis and left‐convex scoliosis, dysmorphic features, and mild developmental delay. Exome sequencing detected the previously unreported *de novo* loss‐of‐function variant in exon 33 of *NOTCH3* p.(Lys2137fs). Following the identification of the gene defect, MRI of the brain and spine revealed temporal encephaloceles, inner ears anomalies, multiple spinal lateral meningoceles, and intra‐ and extra‐dural arachnoid spinal cysts. This case illustrates the power of reverse phenotyping to establish clinical diagnosis and expands the spectrum of clinical manifestations related to LMS to include inner ear abnormalities and multi‐cystic kidney disease.

## 1 INTRODUCTION

Lateral meningocele syndrome (LMS) is an ultra‐rare condition with 22 patients reported to date. Among reported cases, only 8 were shown to carry loss‐of‐function mutations affecting exon 33 of *NOTCH3* (Brown, Gupta, & Sayama, 2017; Ejaz et al., 2016; Gripp et al., 2015). Missense variants located in *NOTCH3* exons 3–11 are responsible for a distinct disorder, namely cerebral arteriopathy, autosomal dominant, with subcortical infarcts and leukoencephalopathy (Joutel et al., 1996). The core clinical manifestation of LMS is lateral spinal meningoceles, occurring with protrusion of the arachnoid and the dura through the intervertebral foramina causing back pain, paresthesia, paraparesis, and neurologic bladder depending on their size and localization. Chiari type 1 malformation, syringomyelia, and hydrocephalus have also been reported. Other features include a recognizable pattern of dysmorphic facial features (low posterior hair line, coarse hair, arched eyebrows, palpebral ptosis, down‐slanting palpebral fissures, hypertelorism, malar hypoplasia, micrognatia, long or smooth philtrum, thin vermillion, high and narrow palate, and cleft palate), low weight, short stature, hypotonia, congenital heart defects, feeding difficulties (dysphagia and gastroesophageal reflux disease), joint laxity, kyphoscoliosis, and vertebral anomalies (Ejaz et al., 2016; Gripp et al., 2015). Cognitive impairment is reported in only one case (Gripp et al., 2015). Mixed or conductive hearing loss was found in three individuals, while ocular anomalies had been rarely reported (Ejaz et al., 2016; Gripp et al., 2015).

We describe a further case with LMS due to a novel *NOTCH3* truncating variant in exon 33 presenting with inner ear abnormalities and cystic kidneys disease that have not been previously reported in LMS.

## 2 CLINICAL REPORT

We report a 7‐year‐and‐8‐month‐old girl who was the second child of healthy and non‐consanguineous parents. Prenatal ultrasound showed bilateral hydronephrosis and oligohydramnios. She was born at term by vaginal delivery complicated by fracture of a clavicle. Her birth weight was 3,660 g. At birth, she developed acute respiratory distress requiring endotracheal intubation. From the first months of life, she had feeding difficulties with frequent regurgitations, failure to thrive, and recurrent episodes of aspiration pneumonia requiring gastrostomy tube. At 2 years, she was diagnosed with severe gastroesophageal reflux disease treated with Nissen fundoplication. Transient hypothyroidism in the first months of life was treated with hormone replacement therapy up to 2 years of age. Bilateral severe neurosensorial hearing loss (80 dB HL) was diagnosed at 6 months of life, and she was treated with hearing aids from the age of 1 year. Postnatal echocardiogram showed patent ductus arteriosus that spontaneously closed, and an atrial septal defect; the ascending aorta was not dilated. She had severe kyphosis and left‐convex scoliosis requiring brace. Multiple vertebral anomalies were detected by X‐ray, including an odontoid process retroflexion with a cleft of anterior arch of C1, cuneal deformation, and posterior scalloping of multiple vertebral bodies. Abdomen ultrasound showed hypoplasia of the left kidney and multiple bilateral renal cysts the largest measuring 3 cm in diameter. She also had microlithiasis and was treated with potassium citrate.

She sat independently at 6 months, walked unassisted at 24 months, and spoke her first words at 12 months. She attended regular school without learning difficulties.

**Figure 1:**
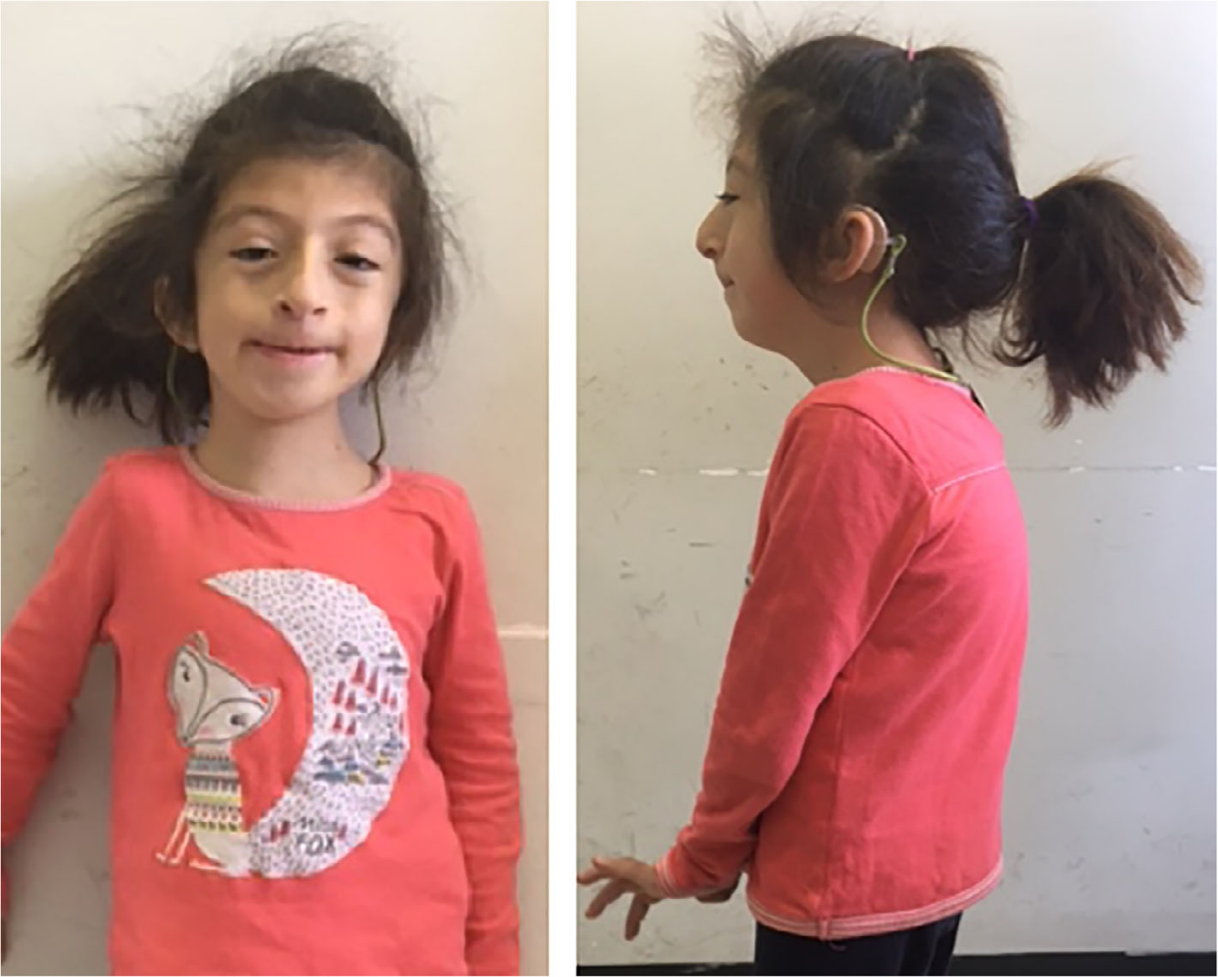
Dysmorphic features of the presented girl including low posterior hair line, arched eyebrows, synophria, bilateral ptosis and down‐slanting palpebral fissures, low‐set ears, anteverted nares, protruding columella, smooth philtrum, thin lips, and micrognatia. Kyphosis is also evident [Color figure can be viewed at http://wileyonlinelibrary.com]

At the age of 5 years, her weight was 19 kg (fourth centile), height 115 cm (fourth centile), and occipitofrontal circumference was 51.5 cm (14th centile), BMI was at the 18th centile. Dysmorphic features included prominence of the occipital bone, low posterior hairline, arched eyebrows, synophria, bilateral ptosis, down‐slanting of the palpebral fissures, low‐set ears, anteverted nares, protruding columella, smooth philtrum, thin lips, micrognatia, and high and narrow palate (Figure 1). She also had joint hypermobility. Array comparative genomic hybridization was normal and *MLL2* and *OFD1* sequencing performed for the suspicion of Kabuki and orofaciodigital syndromes, respectively, did not reveal any pathogenic variants.

Following informed consent, genomic DNA from the patient and her parents underwent exome sequencing (ES), enriched using the SureSelect Clinical Research Exome (Agilent, Technologies, Santa Clara, CA) and sequences in the NextSeq 500 sequencing system (Illumina, San Diego, CA). A custom pipeline based on Burrows‐Wheeler Alignment tool, Genome Analysis Toolkit, and ANNOVAR (Wang et al., 2010) was used to call, annotate, filter, and prioritize variants. TrioES revealed a novel heterozygous *de novo* two‐nucleotide deletion in exon 33 of *NOTCH3* gene (NM_000435: c.6409_6410delTC; p.Lys2137fs).

**Figure 2:**
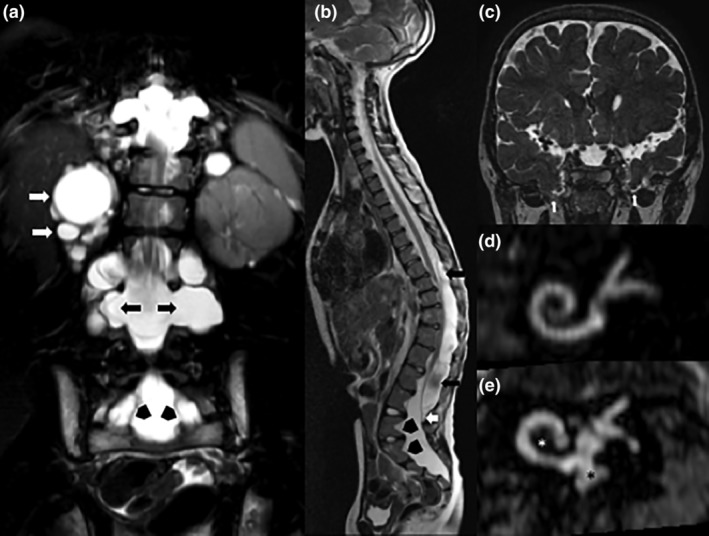
(a) Turbo Spin Echo (TSE) T2‐weighted sequence on coronal plane shows multiple bilateral meningoceles (black arrows), renal cysts (white arrows), and foraminal extra‐dural arachnoid cysts (black arrowheads). (b) TSE T2‐weighted sequence on sagittal plane shows some confluent intra‐dural arachnoid cysts, located posteriorly respect to the spinal cord between T8 and L4‐L5 levels (black arrows). Tethering of the filum terminale at L4‐L5 (white arrow) and posterior scalloping of the lumbar and sacral vertebral bodies (black arrowheads) can also be noted. (c) Driven equilibrium radiofrequency reset pulse (DRIVE) 3D TSE T2‐weighted sequence on coronal plane shows bilateral inferior temporal meningoencephaloceles (white arrows). (d) Oblique coronal multi‐planar reconstructions (MPR) of DRIVE 3D TSE T2‐weighted sequence highlight the differences between a normal internal ear (d) and the inner ear of the subject herein described (e) with hypoplasia of the apical turn of the cochlea (white asterisk), and dysmorphic vestibule (black asterisk)

Brain and spine MRI performed after the identification of *NOTCH3* mutation revealed bilateral meningoencephaloceles of the inferior temporal lobes, multiple spinal extra‐ and intra‐dural arachnoid cysts with tethering of the filum terminale at L4‐L5 and multiple bilateral spinal meningoceles extending from C7 to S3 (Figure 2a–c). MRI of the inner ear showed hypoplasia of the apical turn of the cochlea and of the modiolus, and dysmorphic vestibule (Figure 2d,e).

## 3 DISCUSSION

A total of eight cases with LMS carrying loss‐of‐function variants affecting exon 33 of *NOTCH3* gene have been reported and we herein describe a further LMS individual harboring a novel nonsense variant of *NOTCH3* exon 33. Notably, the presented case had inner ear malformation and cystic kidney disease that have not been previously reported. Interestingly, *Notch3* is expressed in the mouse inner ear (Eddison, Le Roux, & Lewis, 2000) where it supports the differentiation of auditory and vestibular hair cell progenitors (Ma, Rubel, & Raible, 2008). Moreover, Notch signaling has been involved in regeneration of hair cells (Ma et al., 2008; Shu et al., 2019).

The child herein described showed multiple bilateral kidney cysts without liver cysts or fibrosis that have also not been previously reported in LMS. Genes causing dominant and recessive forms of polycystic kidney diseases (*PKD1, GANAB, FCYT, PKD2, DNAJB11*, and *DZIP1L)* had a good coverage in the ES and no pathogenic variants were identified. Disruption of *NOTCH* pathway during development resulted in proximal tubular cysts and, and its overexpression correlated to rapidly growing cysts in kidneys of autosomal recessive and autosomal dominant polycystic kidney disease mouse models (Idowu et al., 2018). Nevertheless, the description of further individuals with LMS is required to conclude whether inner ear malformation and cystic kidney are indeed features of LMS. In conclusion, we describe a further individual with LMS carrying a novel pathogenic variant in *NOTCH3* who presented with inner ear abnormalities and cystic kidney disease that expand the spectrum of phenotypic abnormalities.

## CONFLICT OF INTEREST

The authors declare no conflict of interest.

## AUTHOR CONTRIBUTIONS

G.C., D.A., and N.B.‐P. contributed to conceptualization. M.A., A.T., M.P., and V.N. contributed to methodology. M.P., A.T., V.N., and B.F. contributed to formal analysis. B.C., E.D.G., and A.D. contributed to investigation. G.C. and M.P. contributed to data curation. G.C., D.A., and N.B.‐P. contributed to writing—original draft preparation. G.C. and N.B.‐P contributed to writing—review and editing. N.B.‐P. contributed to supervision.
